# Investigation of Mental Health Literacy and Status of Residents During the Re-Outbreak of COVID-19 in China

**DOI:** 10.3389/fpubh.2022.895553

**Published:** 2022-07-12

**Authors:** Shiming Li, Bingbing Guo, Xiao Lu, Queping Yang, Haohao Zhu, Yingying Ji, Ying Jiang

**Affiliations:** ^1^The Affiliated Wuxi Mental Health Center of Nanjing Medical University, Wuxi Tongren International Rehabilitation Hospital, Wuxi, China; ^2^The Affiliated Wuxi Maternity and Child Health Care Hospital of Nanjing Medical University, Wuxi, China; ^3^Department of Rehabilitation Medicine, The First Affiliated Hospital of Nanjing Medical University, Nanjing, China

**Keywords:** mental health literacy, mental health status, re-outbreak, COVID-19, residents

## Abstract

**Introduction:**

The current field of research on the impact of COVID-19 on mental health was mostly limited to the evaluation of the first round of the epidemic, few reports focused on the impact of the re-emergence of COVID-19. This study aimed to investigate the mental health literacy and status of residents during the re-outbreak of COVID-19 in China.

**Methods:**

The basic information sheet, health literacy survey scale, physical health questionnaire-9 (PHQ-9), generalized anxiety disorder-7 (GAD-7), insomnia severity index (ISI), and Alzheimer dementia 8 (AD8) were applied to evaluate the mental health literacy, mental health status and elderly cognitive function, and χ2 test was applied for analysis of the difference between different groups.

**Results:**

A total of 2,306 participants were involved in this study, of which 734 people completed the mental health literacy survey. The qualified rate of mental health literacy was 6.4%. The difference is statistically significant. A total of 1,015 people completed the survey of mental health status, the prevalence of depressive symptoms was 8.87%, the monthly income of different families (χ2 = 13.96, *P* = 0.01), the self-assessed health status (χ2 = 128.56, *P* < 0.05), the presence or absence of chronic diseases (χ2 = 4.78, *P* = 0.03), among all which the difference was statistically significant; the prevalence of anxiety symptoms was 3.84%, different regions (χ2 = 12.26, *P* < 0.05), occupations (χ2 = 11.65, *P* < 0.05), household monthly income (χ2 = 12.65, *P* = 0.01), self-rated health status (χ2 = 151.11, *P* < 0.05), and chronic diseases (χ2 = 7.77, *P* = 0.01), among all which the differences were statistically significant. The prevalence of insomnia symptoms was 7.98%, different age (χ2 = 18.45, *P* < 0.05), region (χ2 = 5.11, *P* = 0.02), monthly household income (χ2 = 12.68 *P* = 0.01), and self-assessed health status (χ2 = 91.71, *P* < 0.05), in which there was a statistically significant difference between those with or without chronic diseases (χ2 = 3 3.25, *P* < 0.05). A total of 557 elderly people over 65 years old completed the cognitive dysfunction screening, in which the prevalence of cognitive dysfunction was 17.41%, and the difference was statistically significant at the different self-assessed health status (χ2 = 96.24, *P* < 0.05) and with or without chronic diseases (χ2 = 107.09, *P* < 0.05).

**Conclusion:**

The mental health literacy and status of residents have not improved significantly during the second outbreak of the epidemic, indicating that under the normalization of epidemic prevention and control, more attention should be paid to the mental health of residents, and targeted health education and psychological intervention should be carried out to avoid relative adverse events.

## Introduction

The COVID-19 caused by SARS-CoV-2 has spread around the world and the WHO has declared the COVID-19 as a “public health emergency of international concern” (1). The raging virus, the isolation measures adopted by various countries and the interruption of normal life have caused public panic and psychological problems (2–4). A meta-analysis of studies in different countries showed that the average prevalence of depression, anxiety, distress, and insomnia caused by the COVID-19 was 31.4, 31.9, 41.1, and 37.9%, respectively (5). More than half of a total of 1,210 respondents to an online survey conducted in China 2 weeks after the outbreak of COVID-19 rated the psychological impact of the outbreak as moderate or severe, with 36.4% of anxiety, 32.4% of stress, and 31.3% of depression (6); in the follow-up survey after 4 weeks, the levels of depression, anxiety and stress all remained elevated or at the same level (7). An online questionnaire survey in Liaoning Province showed that most of the 263 participants felt the pressure brought about by the epidemic, and more than half of them felt fear and anxiety due to the epidemic (8).

In China, through the joint efforts of the government and the masses, the rebound and import of new cases of COVID-19 have been controlled, and China has entered the stage of normalized epidemic prevention, which is also called the “post-epidemic era.” In the post-epidemic era, although public physical health is gradually recovering, the adverse mental health outcomes caused by the epidemic may still persist or even worsen (9, 10). Studies have shown that psychological problems such as anxiety, depression, and post-traumatic stress disorder (PTSD) are still very common within a period of time after major emergencies such as public health emergencies, and the prevalence of PTSD can even reach 33.3% (11), therefore, more attention should be paid to the public mental health under the post-epidemic era.

Besides, although the prevention and control of COVID-19 in China are in the post-epidemic era, there were outbreaks in some areas from time to time. For example, the COVID-19 outbroke again in Nanjing and Yangzhou, Jiangsu Province in July 2021 (12, 13). Compared with the first-round outbreak of the epidemic in Jiangsu Province, the understanding of the epidemic was more scientific, and the government agencies were more fully prepared for epidemic prevention and control. However, due to the re-emergence of the epidemic, the recovered life and work were disrupted again, with a negative psychological impact on residents, making them feel hopeless, helpless, panic, and even have symptoms such as anxiety, depression, and insomnia (14, 15). The current field of research on the impact of COVID-19 on mental health was mostly limited to the evaluation of the first round of the epidemic, few reports focused on the impact of the re-emergence of the COVID-19. Through a cross-sectional study, the mental health status, and its influencing factors of residents during the re-emergence of the COVID-19 in the Jiangsu Province were discussed, in order to provide a scientific basis for the administrative department to intervene in the mental health impact of the localized COVID-19 outbreak.

## Methods

### Participants

From 15 July 2021 to 20 November 2021, random cluster sampling was applied to determine the three cities from Jiangsu Province as the survey areas. According to the number of permanent residents, each city randomly selected two streets by stratification, three communities (village neighborhood committees) were selected for each street, 56 households were randomly selected in each community, and 1–2 permanent residents over the age of 18 were randomly selected from the household as the survey participants. In this survey, 2,328 questionnaires were distributed, and 2,306 valid questionnaires were recovered, with an effective rate of 99.05%.

### Questionnaire and Evaluation Criteria

The basic information scale, mental health literacy questionnaire, physical health questionnaire-9 (PHQ-9), generalized anxiety disorder-7 (GAD-7), insomnia severity index (ISI), and Alzheimer dementia 8 (AD8) in the “Jiangsu Province Mental Health Promotion Action Baseline Investigation Plan” were applied to investigate the mental health state of the residents. The basic information scale includes the following: gender, age, educational level, marriage, occupation, region, self-reporting chronic diseases (diabetes and hypertension), and other demographic data. The mental health literacy questionnaire is divided into three parts: judgment questions, self-assessment questions, and case analysis questions. There are 20 judgment questions in total, mainly including the common knowledge related to mental health, five points for correct answers, 0 points for wrong answers or unknowing, out of 100 points, of which ten questions (1, 3, 5, 7, 8, 9, 10, 15, 16, 19) aim to express the correct mental health knowledge, the others are wrong mental health knowledge; the second part of the self-assessment questions are mainly about self-behaviors in life and views on mental health, containing a total of eight questions with the total score of 8–32 points; the third part of the case analysis questions is divided into two groups with potential mental health problems, each group contains four questions, with the total score of 40 points; the qualifying criteria of mental health literacy are judgment questions ≥80 points, self-assessment questions ≥24 points, and case analysis questions ≥28 points. PHQ-9 contains nine items, each item scored 0–3, with 5–9 divided into mild depression, 10–14 moderate depression, 15–19 moderately severe depression, and 20–27 severe depression (16). GAD-7 contains seven items, each item is scored by 3-grade, and the higher scores indicated the more severe symptoms (17). There are seven items included in ISI with each item scored from 0 to 4, of which ≥7 indicates the presence of insomnia symptoms (18). AD8 score ≥2 points could be considered cognitive dysfunction (19).

### Investigation Method and Quality Control

The investigation team was composed of public health personnel, mental health prevention and control personnel, and members of the care and support team for the household survey based on the selected list. In terms of quality control, the content of the questionnaire was verified by experts from the Jiangsu Provincial Mental Health Project Working Group, and an implementation plan was issued. Before the survey was carried out, provincial and municipal household survey training was organized. During the investigation, the questionnaires were quality-controlled by two deputy chief physicians of the Wuxi Mental Health Center.

### Statistical Analysis

To establish the database, EpiData 3.1 was used, while SPSS 22 was applied for statistical analysis. Quantitative data were described by M, and qualitative data were described by n (%); the χ2 test was used for the analysis of the difference between different groups. *p* < 0.05 was considered to be statistically significant.

## Results

### Basic Information

A total of 2,328 questionnaires were distributed, 22 unqualified questionnaires were removed, and the final questionnaire effectiveness rate was 99.05%. Among them, there were 738 mental health literacy questionnaires including 4 removed unqualified questionnaires (effective rate 99.46%), of which 49.86% were men (366/734) and 50.14% were women (368/734), aged from 18 to 89 years old, with the median age of 53 years old. There were 1,025 questionnaires on mental health status, containing 10 unqualified questionnaires, with an effective rate of 99.02%, and 51.03% were men (518/1,015) and 48.97% were women (497/1,015), aged 19–92 years old with the median age of 55 years old. The other 565 questionnaires focused on the cognitive dysfunction screening over the age of 65, with the excluded 8 unqualified questionnaires and 98.58% effective rate, with men accounting for 47.94% (267/557) and women accounting for 52.06% (290/557), aged 65–98 years old, and the median age of 85 years old.

### Qualified Rate of Mental Health Literacy

The qualified rate of mental health literacy in this study was 6.40% (47/734). Univariate analysis showed that there were statistically significant differences among different age (χ2 = 23.96, *P* < 0.05) and educational level (χ2 = 23.96, *P* < 0.05), Table 1.

**Table 1:** The mental health literacy of residents.

**Factors**	**No**.	**Qualified number**	**Qualified rate(%)**	***χ^2^***	***P***
**Gender**				0.60	0.44
Male	366	26	7.10		
Female	368	21	5.71		
**Age**				23.96	<0.0001
18–30	49	9	18.37		
31–40	125	14	11.20		
41–50	154	11	7.14		
51–60	172	7	4.07		
>60	234	6	2.56		
**Education level**				24.28	<0.0001
Junior high school and below	404	15	3.72		
High school or secondary school	159	8	5.03		
College	94	11	11.70		
Undergraduate and above	77	13	16.88		
**Area**				0.72	0.40
City	494	29	5.87		
Rural	240	18	7.50		

### Prevalence of Depression and Anxiety Symptoms

The prevalence of depressive symptoms was 8.87% (90/1,015), and the prevalence of anxiety symptoms was 3.84% (39/1,015). The monthly household income (χ2 = 13.96, *P* = 0.01), self-assessed health status (χ2 = 128.56, *P* < 0.05) and the chronic disease (χ2 = 4.78, *P* = 0.03) were statistically significant. Univariate analysis of anxiety symptoms showed that the differences between different regions (χ2 = 12.26, *P* < 0.05), occupation (χ2 = 11.65, *P* < 0.05), monthly household income (χ2 = 12.65, *P* = 0.01), self-assessed health status (χ2 = 151.11, *P* < 0.05), and the presence of chronic diseases (χ2 = 7.77, *P* = 0.01) were statistically significant, Table 2.

**Table 2:** The mental health status of residents.

**Factors**	**No**.	**Depression**				**Anxiety**			
		**No**.	**Rate (%)**	***χ^2^***	***P***	**No**.	**Rate (%)**	***χ^2^***	***P***
**Gender**				0.18	0.67			0.01	0.98
Male	518	34	6.56			20	3.86		
Female	497	56	11.27			19	3.82		
**Age**				6.75	0.15			1.98	0.74
18–30	63	10	15.87			1	1.59		
31–40	150	12	8.00			4	2.67		
41–50	189	13	6.88			9	4.76		
51–60	230	16	6.96			9	3.91		
>60	383	39	10.18			16	4.18		
**Education level**				2.47	0.48			7.62	0.05
Junior high school and below	159	15	9.43			12	7.55		
High school or secondary school	410	36	8.78			13	3.17		
College	236	16	6.78			9	3.81		
Undergraduate and above	210	23	10.95			5	2.38		
**Area**				2.86	0.09			12.26	<0.0001
City	679	53	7.81			16	2.36		
Rural	336	37	11.01			23	6.85		
**Profession**				8.04	0.15			11.65	0.04
Professionals & technical	155	16	10.32			5	3.23		
Business/service	98	12	12.24			3	3.06		
Farmer	98	7	7.14			5	5.10		
Worker	402	25	6.22			9	2.24		
Retire	45	6	13.33			5	11.11		
Other	217	24	11.06			12	5.53		
**Marital status**				0.90	0.64			0.65	0.72
Unmarried	52	6	11.54			1	1.92		
Married	899	77	8.57			35	3.89		
Divorced/widowed	64	7	10.94			3	4.69		
**Chronic disease**				4.78	0.03			7.77	0.01
No	584	42	7.19			14	2.40		
Yes	431	48	11.14			25	5.80		
**Monthly household income (yuan)**				13.96	0.01			12.65	0.0073
0–1499	64	7	10.94			1	1.56		
1,500–2,999	171	27	15.79			13	7.60		
3,000–4,999	378	29	7.67			10	2.65		
5,000–7,999	284	21	7.39			14	4.93		
>8,000	118	6	5.08			1	0.85		
**Drinking**				1.12	0.29			0.93	0.34
No	838	78	9.31			30	3.58		
Yes	176	12	6.82			9	5.11		
**Smoking**				0.64	0.43			0.55	0.46
No	778	66	8.48			28	3.60		
Yes	236	24	10.17			11	4.66		
**Self-assessed health status**				128.56	<0.05			151.11	<0.0001
Good	354	14	3.95			5	1.41		
Better	345	18	5.22			4	1.16		
General	284	40	14.08			17	5.99		
Relative poor	27	14	51.85			10	37.04		
Poor	4	4	100.00			3	75.00		

### Prevalence of Insomnia Symptoms

The prevalence of insomnia symptoms in this study was 7.98% (81/1,015). The univariate analysis of insomnia symptoms showed that the differences between different age (χ2 = 18.45, *P* < 0.05), region (χ2 = 5.11, *P* = 0.02), monthly household income (χ2 = 12.68 *P* = 0.01), self-assessed health status (χ2 = 91.71, *P* < 0.05), and the presence of chronic diseases (χ2 = 33.25, *P* < 0.05) were statistically significant, Table 3.

**Table 3:** The status of insomnia among residents.

**Factors**	**No**.	**No. of insomniacs**	**Rate (%)**	***χ^2^***	***P***
**Gender**				0.60	0.44
Male	518	38	7.34		
Female	497	43	8.65		
**Age**				18.45	<0.0001
18–30	63	2	3.17		
31–40	150	3	2.00		
41–50	189	12	6.35		
51–60	230	18	7.83		
>60	383	46	12.01		
**Education level**				13.56	<0.0001
Junior high school and below	159	21	13.21		
High school or secondary	410	34	8.29		
school					
College	236	20	8.47		
Undergraduate and above	210	6	2.86		
**Area**				5.11	0.02
City	679	45	6.63		
Rural	336	36	10.71		
**Profession**				9.89	0.08
Professionals & technical	155	11	7.10		
Business/service	98	7	7.14		
Farmer	98	13	13.27		
Worker	402	24	5.97		
Retire	45	7	15.56		
Other	217	19	8.76		
**Marital status**				2.85	0.24
Unmarried	52	1	1.92		
Married	899	74	8.23		
Divorced/widowed	64	6	9.38		
**Chronic disease**				33.25	<0.0001
No	584	22	3.77		
Yes	431	59	13.69		
**Monthly household**				12.68	0.01
**income (yuan)**					
0–1499	64	8	12.50		
2,500–2,999	171	23	13.45		
3,000–4,999	377	28	7.43		
5,000–7,999	284	17	5.99		
>8,000	118	5	4.24		
**Drinking**				2.28	0.13
No	838	62	7.40		
Yes	176	19	10.8		
**Smoking**				0.75	0.39
No	778	59	7.58		
Yes	236	22	9.32		
**Self-assessed health status**				91.71	<0.0001
Good	354	11	3.11		
Better	345	19	5.51		
General	284	36	12.68		
Relative poor	27	13	48.15		
Poor	4	2	50.00		

### Prevalence of Cognitive Dysfunction in the Elderly Over 65 Years Old

The prevalence of cognitive dysfunction in the elderly over 65 years old was 17.41% (97/557). Univariate analysis showed that the self-assessed health status (χ2 = 96.24, *P* < 0.05) and the presence of chronic diseases (χ2 = 107.09, *P* < 0.05) were significantly associated with cognitive dysfunction, Table 4.

**Table 4:** The cognitive dysfunction of residents over 65 years old.

**Factors**	**No**.	**No. with cognitive impairment**	**Rate (%)**	***χ^2^***	***P***
**Gender**				2.11	0.15
Male	267	53	19.85		
Female	290	44	15.17		
**Age**				7.24	0.12
65–70	267	53	19.85		
71–80	204	26	12.75		
>80	86	18	20.93		
**Education level**				2.47	0.48
Junior high school and below	226	60	26.55		
High school or secondary school	158	13	8.23		
College	138	16	11.59		
Undergraduate and above	35	8	22.86		
**Area**				1.63	0.20
City	377	71	18.83		
Rural	180	26	14.44		
**Marital status**				3.58	0.62
Unmarried	12	4	33.33		
Married	358	73	20.39		
Divorced/widowed	187	20	10.70		
**Chronic disease**				107.09	<0.0001
No	367	20	5.45		
Yes	190	77	40.53		
**Self-assessed health status**				96.24	<0.0001
Good	101	23	22.77		
Better	236	38	16.10		
General	184	20	10.87		
Relative poor	28	12	42.86		
Poor	8	4	50.00		

## Discussion and Conclusion

The outbreak of the emergent public health event of COVID-19 has brought a huge impact on the development of society, not only on the economy and development of the society but also on the mental health of the residents. With the joint efforts of the whole society, the epidemic has been effectively controlled, the order of the whole society has gradually recovered, and the public's psychological condition has also been eased with the effective prevention and control of the epidemic. However, the re-outbreak of the epidemic in China caused the entire city to be shut down again, and the psychological state of the public was affected again with even more helpless and desperate than the first time, which caused more serious psychological problems. In the background of the outbreak of epidemics in Nanjing and Yangzhou, Jiangsu Province, this study conducted a survey on the mental health status of residents in Jiangsu. Through the self-made mental health literacy questionnaire, a mental health literacy survey was carried out in Jiangsu and found that the qualified rate of mental health literacy was 6.40%, which was significantly lower than the 69.50% qualified rate of the residents' mental health literacy survey carried out in Guiyang in 2021 (20). Residents with younger age and higher education have a more comprehensive understanding of mental health knowledge, a wider range of ways to accept mental health-related knowledge, and easier acceptance and understanding of mental health-related knowledge. The results indicated that the mental health literacy of residents has not been alleviated due to the understanding of the epidemic, scientific prevention and control measures, and the passage of time during the re-outbreak of COVID-19, therefore, there is still an urgent need to pay attention to and intervene in the mental health of residents.

The results of the mental health status of residents showed that the prevalence of depressive symptoms was 8.87%, which was lower than that during the first-round outbreak of COVID-19 with the prevalence of moderately and severely depressive symptoms of 17.47% (3), but the prevalence of depressive symptoms was higher than the 6.8% reported in domestic surveys (8). In terms of depression, residents with different monthly household incomes have different rates of depressive symptoms, higher household incomes mean less impact from the pandemic followed by restrictions on travel and other epidemic prevention, which was consistent with the study (21) found that people with higher household incomes had lower levels of depression. Domestic research (22) found that the detection rate of depressive symptoms in patients with chronic diseases was as high as 44.37%, which was significantly higher than that of ordinary residents. In terms of self-rated health, the prevalence of depressive symptoms was also different, and lower health assessments would lead to poor mental health, resulting in depression and anxiety. The survey found that the prevalence of anxiety symptoms was 3.84%, and the prevalence of anxiety symptoms was lower than the domestic report of 7.60% (7). Studies (23, 24) have revealed that the prevalence of anxiety in rural areas was higher than that in cities, and different occupations will affect the occurrence of anxiety, which was consistent with the results of this study. Different monthly household incomes (25, 26), self-assessed health status, and residents with or without chronic disease were found associated with the occurrence of anxiety symptoms.

This study found that the prevalence of insomnia among residents was 7.98%, which was lower than 23.26% in Beijing (27) and 22.3% in Gansu Province (28). The prevalence of insomnia was different in different regions, age, monthly household income, self-assessed health status, chronic diseases, and other characteristics, which was consistent with other studies, in which lower age and higher monthly household income were the protection factor of insomnia (27, 29). In terms of the cognitive dysfunction in the age of 65 and above, this paper found that the prevalence of cognitive dysfunction in the elderly was 17.41%, which was lower than 20.14% in Xiamen (30) and 18.59% in Nanchang (31), higher than that in Zhangjiakou (12.2%) (32). There were significant differences in the prevalence of cognitive dysfunction among the elderly with different self-rated health statuses and chronic diseases, which were consistent with reported studies that elderly people with stroke and other chronic diseases, or perceived poor health status were more likely to have cognitive impairment (32, 33).

In this study, under the normalization of epidemic prevention and control, mental health literacy and the status survey were conducted among residents in local outbreak areas to better evaluate the mental health problems of residents in relatively developed areas under the repeated impact of the epidemic, but there are also some limitations. The sample size is relatively small, and the impact of other recent life emergency events on the psychology of the respondents has not been fully evaluated. Further investigation in public psychology after the epidemic is still urgently needed.

To sum up, the residents in Jiangsu have a low pass rate of mental health literacy, and the problems of mental health and cognitive dysfunction of the elderly are relatively prominent under the normalization of epidemic prevention and control. It is recommended to carry out targeted health education and mental health services and formulate spiritual health service policies to prevent or mitigate serious mental health problems in the community under COVID-19.

## Data Availability Statement

The dataset generated and analysed during the current study could be available from the corresponding author on reasonable request.

## Ethics Statement

The studies involving human participants were reviewed and approved by Wuxi Mental Health Center. The patients/participants provided their written informed consent to participate in this study.

## Author Contributions

SL and HZ conceived the study. BG, QY, and YJ performed a survey and summary. YJ, XL, and HZ wrote the manuscript. All authors contributed to the article and approved the submitted version.

## Funding

The work was supported by the National Natural Science Foundation of China (No. 8210131157), Wuxi Municipal Health Commission (Nos. Q202050, Q202101, Q202167, M202167, and ZH202110), Wuxi Taihu Talent Project (Nos. WXTTP2020008 and WXTTP2021), High-level talent training project of Wuxi Taihu Talent Plan (HB2020071), Wuxi Medical Development Discipline Project (No. FZXK2021012), and Jiangsu Research Hospital Association for Precision Medication (JY202105).

## Conflict of Interest

The authors declare that the research was conducted in the absence of any commercial or financial relationships that could be construed as a potential conflict of interest.

## Publisher's Note

All claims expressed in this article are solely those of the authors and do not necessarily represent those of their affiliated organizations, or those of the publisher, the editors and the reviewers. Any product that may be evaluated in this article, or claim that may be made by its manufacturer, is not guaranteed or endorsed by the publisher.
